# Effects of Core Training in Physical Fitness of Youth Karate Athletes: A Controlled Study Design

**DOI:** 10.3390/ijerph19105816

**Published:** 2022-05-10

**Authors:** Menderes Kabadayı, Soner Karadeniz, Ali Kerim Yılmaz, Emre Karaduman, Özgür Bostancı, Zeki Akyildiz, Filipe Manuel Clemente, Ana Filipa Silva

**Affiliations:** 1 Faculty of Yaşar Doğu Sport Sciences, Ondokuz Mayıs University, Samsun 55270, Turkey; menderes@omu.edu.tr (M.K.); karadenizsoner@msn.com (S.K.); akerim.yilmaz@omu.edu.tr (A.K.Y.); emre.karaduman@omu.edu.tr (E.K.); bostanci@omu.edu.tr (Ö.B.); 2 Faculty of Sport Sciences, Gazi University, Ankara 06500, Turkey; zekiakyldz@hotmail.com; 3 Escola Superior Desporto e Lazer, Instituto Politécnico de Viana do Castelo, Rua Escola Industrial e Comercial de Nun’Álvares, 4900-347 Viana do Castelo, Portugal; anafilsilva@gmail.com; 4 Research Center in Sports Performance, Recreation, Innovation and Technology (SPRINT), 4960-320 Melgaço, Portugal; 5 Instituto de Telecomunicações, Delegação da Covilhã, 1049-001 Lisboa, Portugal

**Keywords:** karate, strength training, core training, athletic performance

## Abstract

This study aimed to analyse the impact of an 8-week core strength training (CST) programme in youth karate practitioners on core endurance, agility, flexibility, sprinting, jumping, and kick performance. This study followed a randomized parallel design. Twenty-nine participants (age: 12.86 ± 0.81 years old; height: 152.55 ± 10.37 cm; weight: 42.93 ± 8.85 kg) were allocated to a CST programme (*n* = 16) performed thrice weekly or to a control group (*n* = 13) only performing the sport-specific (karate) training. Participants were assessed three times (baseline, mid and post-intervention) for the following tests: (i) flexor endurance test (FET); (ii) back extensor test (BET); (iii) lateral musculature test (LMT); (iv) flexibility; (v) chance of direction (COD); (vi) countermovement jump (CMJ); (vii) back muscle strength (BMS); (viii) horizontal jump (LJ); (ix) sprint test; and (x) karate kick test (KKT). Between-group analysis revealed significant advantages for the CST group on the FET (*p* < 0.001), BET (*p* < 0.001), LMT (*p* < 0.001), 20 m sprint (*p* = 0.021) and KKT for right (*p* < 0.006) and left (*p* < 0.020) legs. No significant differences were found between groups in the remaining physical fitness variables (*p* > 0.05). The within-group changes revealed significant improvements in the CST group at flexibility (*p* = 0.002), COD (*p* < 0.001), CMJ (*p* < 0.001), BMS (*p* < 0.002), 20 m sprint (*p* = 0.033), and KKT (*p* < 0.001). In addition, within-group changes in the control group were also significant in flexibility (*p* = 0.024) and right kick (*p* < 0.042). We conclude that the CST programme improves core endurance and karate kick performance; however, it is not effective enough for other physical performance parameters in KR practitioners.

## 1. Introduction

Karate (KR) is a martial art which consists of fast repetitions and specific defensive sequences. This sport, which also means “empty hands”, is known as a martial art that is applied without using any kind of weapon to the opponent [1,2]. This martial art, which is performed in two ways called kumite and kata, is a combination of dynamic and coordinated moves that require the practitioner to give multiple reactions quickly, to attack simultaneously, and to have highly functional activity and advanced biological adaptation [3,4]. Researchers have reported that KR is a martial art in which motor and functional skills such as muscle strength, flexibility, speed, agility, balance, reaction time, coordination should be at a high level [5,6,7]. In terms of physiology, KR practitioners should have a high level of cardiorespiratory endurance, muscular endurance and anaerobic capacity. KR is a martial art that does not mainly depend on only one performance parameter. It requires many physical and physiological parameters in combination for success, as in all martial arts [8,9]. In general, researchers define successful KR practitioners as individuals with a high fitness level [10]. 

KR includes moves such as kicking, throwing, and jumping; thus, it requires absolute muscle strength and power to achieve a high level of performance and to meet the increasing strength demand [2]. Performing maximal muscle strength and power in the best way occurs through successful stability and balance in all martial arts [11,12]. Accordingly, some training methods need to provide stimulation for the development of the components of fitness and to create a situation to develop body balance and stability in all sports branches [2]. In recent years, the development and significance of KR have encouraged researchers to investigate the effects of various training protocols on this martial art [13,14,15]. Strength and conditioning specialists, coaches, and scientists working on training have developed a large number of methods to understand the effects of these on KR performance and to reach success by trying different and advanced training protocols.

Studies conducted have clearly emphasized that core strength is vitally important in KR practitioners since they require good karateka balance and stability during punch and kick moves [16,17]. The core has been defined as a muscular corset with abdominals anteriorly, erector spinae and gluteals posteriorly, and the diaphragm as a roof. Additionally, core stabilization has been defined as the stabilization of the body centre against dynamic movements of the limbs and the absorption of pressure on the core of the body [18,19]. Core strength training (CST) aims to make a balanced distribution of strength to the body, creating strength balance. Furthermore, it provides neuromuscular control in the muscle system in a balanced way [20]. With these aspects, CST has become important in increasing physical fitness and athletic performance [21]. Since related studies have considered core areas as the centre of the kinetic chain in exercise and sport activities, they have stated that strength, balance and movement control of this area may maximize lower and upper extremity functions [21,22]. There are also studies in the literature which show that CST protocols positively affect certain performance components such as flexibility, agility and respiratory function [23,24,25]. It is thought that core strength may have a positive affect not only on sport-specific skills of KR, but also on some other performance components such as speed, agility, flexibility, and endurance. A study investigating the 10-week-long effects of CST in karate practitioners has shown that it improves balance, reaction time, strength and some karate-specific techniques [26]. In addition, studies conducted on different sports branches have clearly shown that CST has a positive effect on performance components, and it even decreases predisposition to injury [27,28,29,30,31,32]. 

There are a limited number of studies in the literature investigating the effects of CST on performance components and core stabilization in karate practitioners. When evaluated in this respect, the present study is the first to investigate the effects of 8-week CST on agility, speed, strength, anaerobic strength, flexibility and the number of kicks together in young karate practitioners. In addition, our present study is valuable in terms of examining the chronic effects of core strength training on these performance parameters, which are important for karate players. Our study hypothesizes that CST will improve the components of all these performance components. 

## 2. Materials and Methods

### 2.1. Study Design

This study was designed as a randomized parallel controlled trial. Before the trial was initiated, the participants were randomly assigned to one of the two groups as control (*n* = 16) and experimental (*n* = 16) using a computer-generated simple randomization procedure [33]. Participants in the control group were included in the sport-specific (karate) programme (SSp), while those in the experimental group were included in the CST programme in addition to the SSp. The regular karate practice occurred for approximately 45–60 min 5 times per week, for 8 weeks.

The SSp included kicking, punching, striking, and blocking moves in both stationary and variable positions. The CST included 9 exercises: (1) push up, (2) abdominal crunch, (3) vertical leg crunch, (4) jack knife, (5) supine knee drop side-to-side, (6) reverse crunch, (7) superman, (8) cat–camel stretch, and (9) plank jack (Table 1). Participants completed a 10 min warm-up including several practice trials before testing. The CST was performed for approximately 30–35 min three times per week for 8 weeks [34]. Both testing sessions took place between 2–3 p.m.

All participants were tested 3 times as (a) before CST, (b) 4 weeks after CST and (c) 8 weeks after CST, and they visited the laboratory (total visit 6) twice for each test period (Table 2). In these visits, flexibility, agility, countermovement jump, back muscle strength, horizontal jump, sprint (10 m and 20 m), kick and core endurance (flexion, extension and lateral) performances including physical fitness parameters were recorded. Participants were allowed at least two familiarization trials to feel comfortable with the tests and to perform them correctly. They were also requested to continue their normal diet, to consume a light meal at least 2 h before each trial, and to not perform any intense physical activity in the previous 24 h before the test. All measurement trials were conducted by the same researchers at the same time of day (±1 h).

### 2.2. Setting

The study was carried out on a team affiliated with the Turkish Karate Federation between April–June 2021. None of the athletes participated in national or international competitions during the study process. The CST intervention lasted for 8 weeks, on Tuesdays, Thursdays, and Saturdays during the weeks with laboratory visits, and on Monday, Wednesday, and Friday during the other weeks.

### 2.3. Participants

A total of 32 karate practitioners, 17 female and 15 male, from the same club, participated in the study; however, participants (male) corresponding to three controls (19%) were excluded for personal reasons, and the study was continued with a total of 29 participants in Table 3. The criteria for inclusion was to have 4 years of training experience in karate with regular training sessions 4–5 times per week, and no orthopaedic or cardiorespiratory contraindications, or any restrictions. The athletes’ mean ± SD age, height, weight were: 12.86 ± 0.81 years, 152.55 ± 10.37 cm, and 42.93 ± 8.85 kg, respectively. All athletes and families were fully informed about the possible problems related with the experimental procedures. The study was approved by the Clinical Research Ethics Committee at the University of Ondokuz Mayıs (approval number: 2022000043) and was conducted according to the Declaration of Helsinki [35]. All parents provided written informed consent.

### 2.4. Procedures

#### 2.4.1. Context of Data Collection

**Scheme 1:**
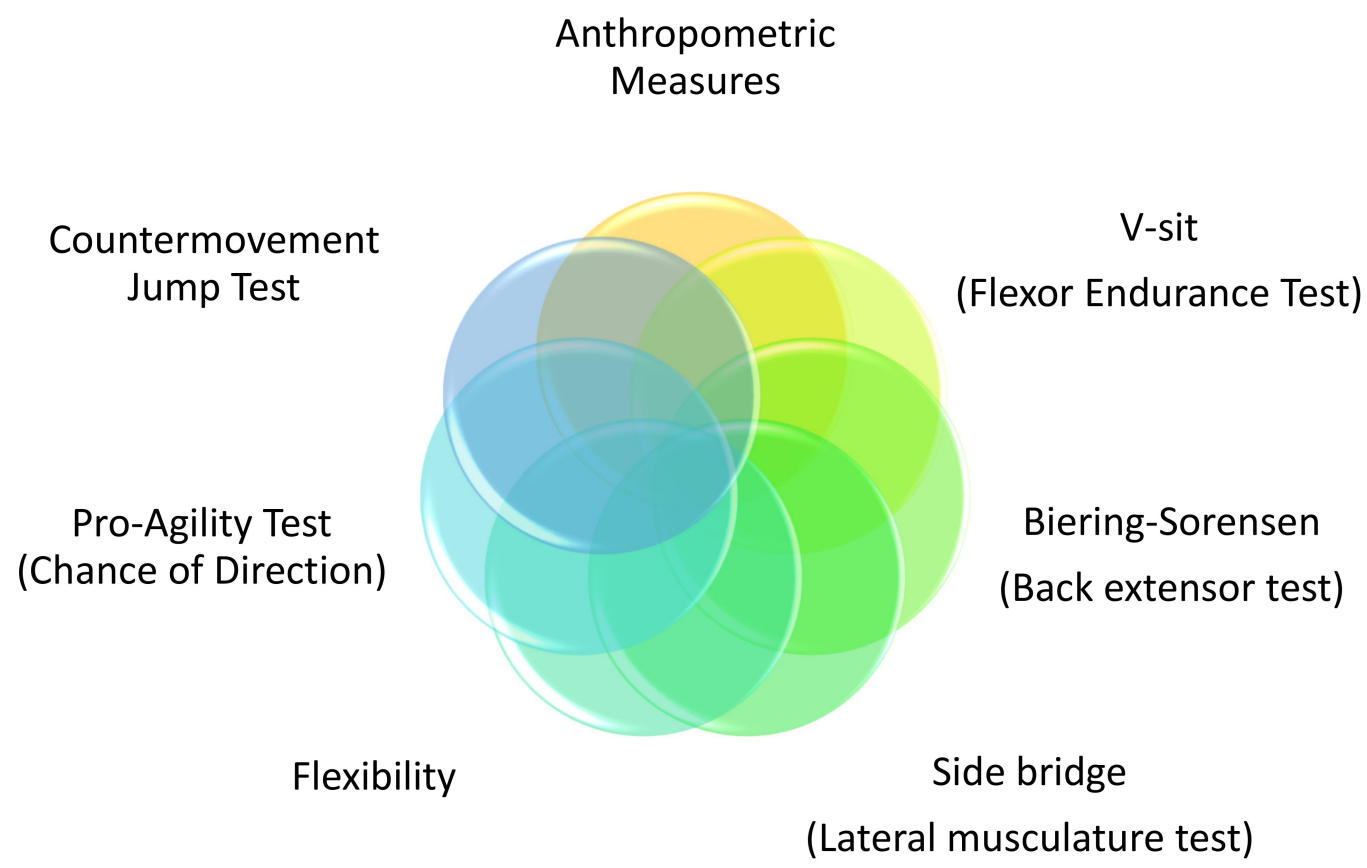
Performance measurement tests applied to athletes.

Each of the assessments occurred 1 day after the last training sessions. The assessment process started at 1 p.m. On day 1 of the assessments, the following tests were made: anthropometric measures (first visit only), flexibility, countermovement jump, horizontal jump and core endurances (flexion, extension and lateral). On day 2, the following tests were made: pro-agility, back muscle strength, sprints and kick test. The data collection process of the athletes is shown in Scheme 1. A rest period of 5 min was provided between tests. Participants dressed in the same shorts, t-shirts, and sneakers (~0.3 kg) in the assessments.

#### 2.4.2. Anthropometric Measures

A calibrated electronic scale (SECA, Hamburg, Germany) was used to assess body mass (with sports clothes without shoes) and height (anatomical position) (SECA, Hamburg, Germany). Measurements were recorded to precision of 0.01 kg and 0.1 cm, respectively. 

#### 2.4.3. V-Sit—Flexor Endurance Test

The flexor endurance test (FET) included the adaptation of a sit-up position with the back resting against a jig angled at 55° flexion, the hips and knees at 90° flexion, and the arms folded across the chest. The test was started when the jig was pulled from the back and ended when a neutral spine posture could no longer be maintained. The feet were fixed throughout the test [34]. Each participant performed a total of 2 trials, one of which was a familiarization trial, with a 60–90 s rest period. Test time was recorded in seconds using a stopwatch. This test was applied to evaluate the core endurance of the subjects.

#### 2.4.4. Biering–Sorensen—Back Extensor Test

The Biering–Sorensen extension test (BET) included stabilization of the pelvis, hips and knees by extending the upper part of the body to the tip of a bank (examination plinth). While the body position was kept as long as possible, the hands were kept crossed on the chest and on opposite shoulders. The test was ended when the flat position could not be maintained. The legs were kept as straight as possible during the test [34,36]. Each participant performed a total of 2 trials, one of which was a familiarization trial, with a 60–90 s rest period. Test time was recorded in seconds using a stopwatch. This test was applied to evaluate the core endurance of the subjects.

#### 2.4.5. Side Bridge—Lateral Musculature Test

The side bridge test (LMT) included participants supporting themselves in a sidelying position with one elbow and feet while lifting their hips from the floor to create a smooth line down their body length (top foot was placed in front of bottom). Knees were on the extensions in line with the feet. The uninvolved arm was held across the chest with the hand placed on the opposite shoulder. The test was ended when the sidelying position was disrupted or when the hip fell down. This test was performed on both the right and left sides [34]. Each participant performed a total of 2 trials, one of which was a familiarization trial, with a 60–90 s rest period. Test time was recorded in seconds using a stopwatch. This test was applied to evaluate the core endurance of the subjects.

#### 2.4.6. Flexibility

Flexibility measurements were performed by using Baseline Sit and Reach Trunk Flexibility Box (Baseline Evaluation Instruments, New York, NY, USA). The participants were asked to rest their feet on the box in the long sitting position with their trunks flexed to 90°. They placed both hands on the trunk flexion meter and flexed forward slowly. The measurement was made twice (2 min of rest between trials), and the best score was recorded in centimetres.

#### 2.4.7. Pro-Agility Test (Chance of Direction)

Pro-agility (COD) test started in front of an electronic timing gate between two lines placed with equal distance to an area of 10 yard (9.14 m) long. The participants turned and ran to the side they chose (4.57 m) and touched the line with their hands (i). After this, they turned 180° and ran for 9.14 m to the opposite side (the other line) and touched the line with their hands (ii). Finally, they turned 180° again, and they ran to the starting line and finished the test running (4.57 m) (iii) [37]. Photocells were adjusted according to the hip alignment of the participants. The participant started 0.3 m before the first pair of photocells. The participants started at split or foot parallel position. Participants performed 2 trials. They were asked to always use the same foot for the braking phase. The fastest time of trials was considered for analysis with the name COD time(s). This test was applied to evaluate the agility of the subjects.

#### 2.4.8. Countermovement Jump Test

Countermovement jump (CMJ) test was performed with a Jump Meter (Vertical Jump Meter T.K.K. 5406, Takei Co., Niigata, Japan) [38] which recorded jump height. The participants performed all their jumps by placing their hands on their hips and by using the squatting depth they chose (knee flexion ≤ 90°). Each participant performed the jump twice (2 min of rest between trials), and the best score was recorded. The measured obtained for further data treatment was the CMJ height in centimetres (cm). This test was applied to determine the anaerobic power of the subjects.

#### 2.4.9. Back Muscle Strength

Back muscle strength (BMS) was determined from the maximal isometric strength of the trunk muscles in a standing position with 30° lumbar flexion via a digital back muscle strength meter (T.K.K. 5402, Takei Co., Niigata, Japan) [39]. Each participant performed two trials involving explosive power, and the best score was recorded. The measure obtained for data treatment was BMS, measured in kilograms. 

#### 2.4.10. Horizontal Jump

Standing long jump (LJ) was used to predict horizontal jump performance. Participants stood behind a line marked on the floor with their feet slightly parted. They were asked to jump forward as much as possible by bending their knees and swaying their arms forward. In all trials, they were asked to have parallel feet at the moment of jumping and landing. The jumping line and the closest landing touch point (the back of heels) was recorded as the jumping distance. Each participant performed two trials (2 min of rest between trials), and the best score was recorded. The measure obtained for further data treatment was LJ, measured in meters. This test was applied to determine the anaerobic power of the subjects.

#### 2.4.11. Sprint Test

Linear sprint times of the participants were determined by using photocell gates (Newtest 2000) placed at distances of 10 and 20 m. The photocell gates were positioned 10 and 20 m from a predetermined starting point. The participants were told to run as fast as possible for 20 m distance starting from the standing point. Photocell heights were adjusted according to the participant’s hip height. The participant started 0.7 m before the first pair of photocells. The tests started in a split position of the foot always with the same preferred leg. Participants performed 2 trials. Each participant made two trials with 3 min of passive resting, and their best result was recorded in seconds. This test was performed to determine the speed and acceleration of the subjects. 

#### 2.4.12. Kick Performance Test 

The kick performance test (KKT) of the participants was evaluated using the Mawashi Geri kick technique. The roundhouse kick to the opponent’s head (Mawashi-Geri) is the most commonly used kicking technique in karate [40]. The roundhouse kick is circular and attacks the opponent from the side. The kick was executed with the rear leg and was recorded at one height from knee to hip [41]. Participants were in combat position and aimed to touch a specific area of a previously marked plate. Before starting the trials, the starting position, the distance, and the height of the kicking plate were adjusted according to the anthropometric characteristics of each participant. All participants were instructed to kick the most optimally designated area repeatedly for 15 s. The kick performance included the total number of kicks scored at the end of the test. Performance was recorded for both feet as repetitions (rep).

### 2.5. Statistical Procedures

The normality of the data was tested with the Shapiro–Wilk test. It was determined that all values showed normal distribution. A mixed ANOVA (factor*time) was used to control the within-group and intra-group analysis for the performance tests values. Group time interaction values were reported as *p* value and partial eta squared. Within-group variations at the pre-, middle, and post-interventions were tested by repeated measures ANOVA test. Differences between groups were tested using the independent t test. Magnitude of differences was tested using the standardized effect size of Cohen [1]. The magnitude of differences was interpreted using the following thresholds [1]: [0.0;0.2], trivial; [0.2;0.5], small; [0.5;0.8], medium; >0.8, large. All analyses were performed via R version R 4.1.2. In this study, the sample size was tested with g power analysis at the beginning of the study. G power was determined with α at 0.05 and 1-β error probability is 0.85 for the 26 participants. We performed an a priori estimation of power and sample size through the G-Power software (version 3.1.9.6) programme written by Kiel University, made in Germany. 

## 3. Results

Descriptive statistics of pre- and post-test values, within-group and between-group analyses can be found in Table 1. Significant interactions were found between groups and time (pre-post) in the mixed ANOVA conducted for FET (*p* = ≤0.001; $\eta_{p}^{2}$ ≤ 0.340), BET (*p* = ≤0.001; $\eta_{p}^{2}$ ≤ 0.256), right LMT (*p* = ≤0.001; $\eta_{p}^{2}$ ≤ 0.340), left LMT (*p* = ≤0.001; $\eta_{p}^{2}$ ≤ 0.239), and Right kick (*p* = 0.001; $\eta_{p}^{2}$ ≤ 0.156). No significant interactions between groups and time (pre-post) were found in mixed ANOVA for the case of flexibility (*p* = 0.289; $\eta_{p}^{2}$ ≤ 0.030), Pro-agility (*p* = 0.355; $\eta_{p}^{2}$ ≤ 0.025), CMJ (*p* = 0.501; $\eta_{p}^{2}$ ≤ 0.017), back strength (*p* = 0.245; $\eta_{p}^{2}$ ≤ 0.034), long jump (*p* = 0.975; $\eta_{p}^{2}$ ≤ 0.007), 10 m time (*p* = 0.270; $\eta_{p}^{2}$ ≤ 0.032), 20 m time (*p* = 0.391; $\eta_{p}^{2}$ ≤ 0.023), left kick (*p* = 0.032; $\eta_{p}^{2}$ ≤ 0.082). Table 4 presents descriptive statistics within and between groups.

**Figure 1:**
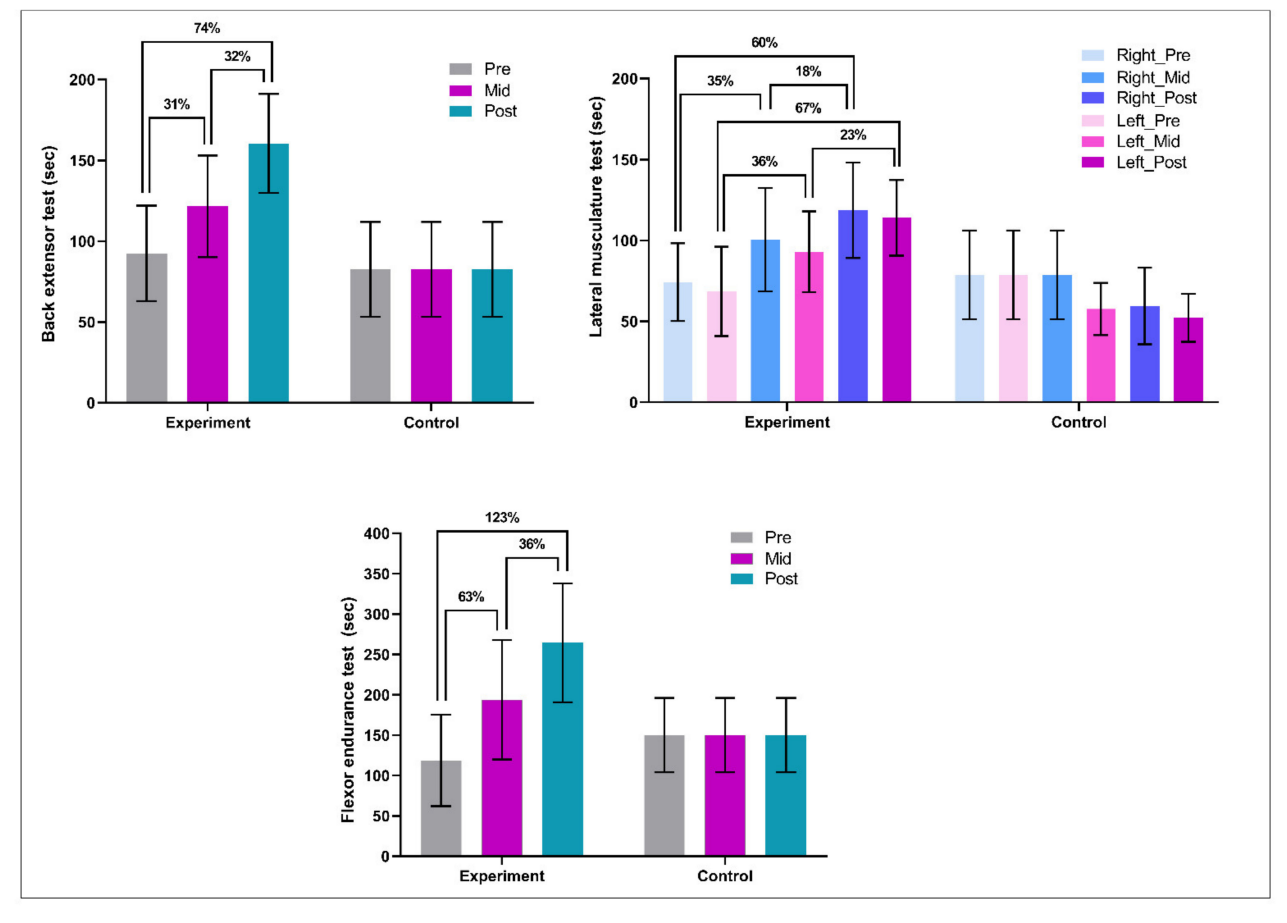
Descriptive statistics, within-group variations of means of FET, BET and LMT parameters.

**Figure 2:**
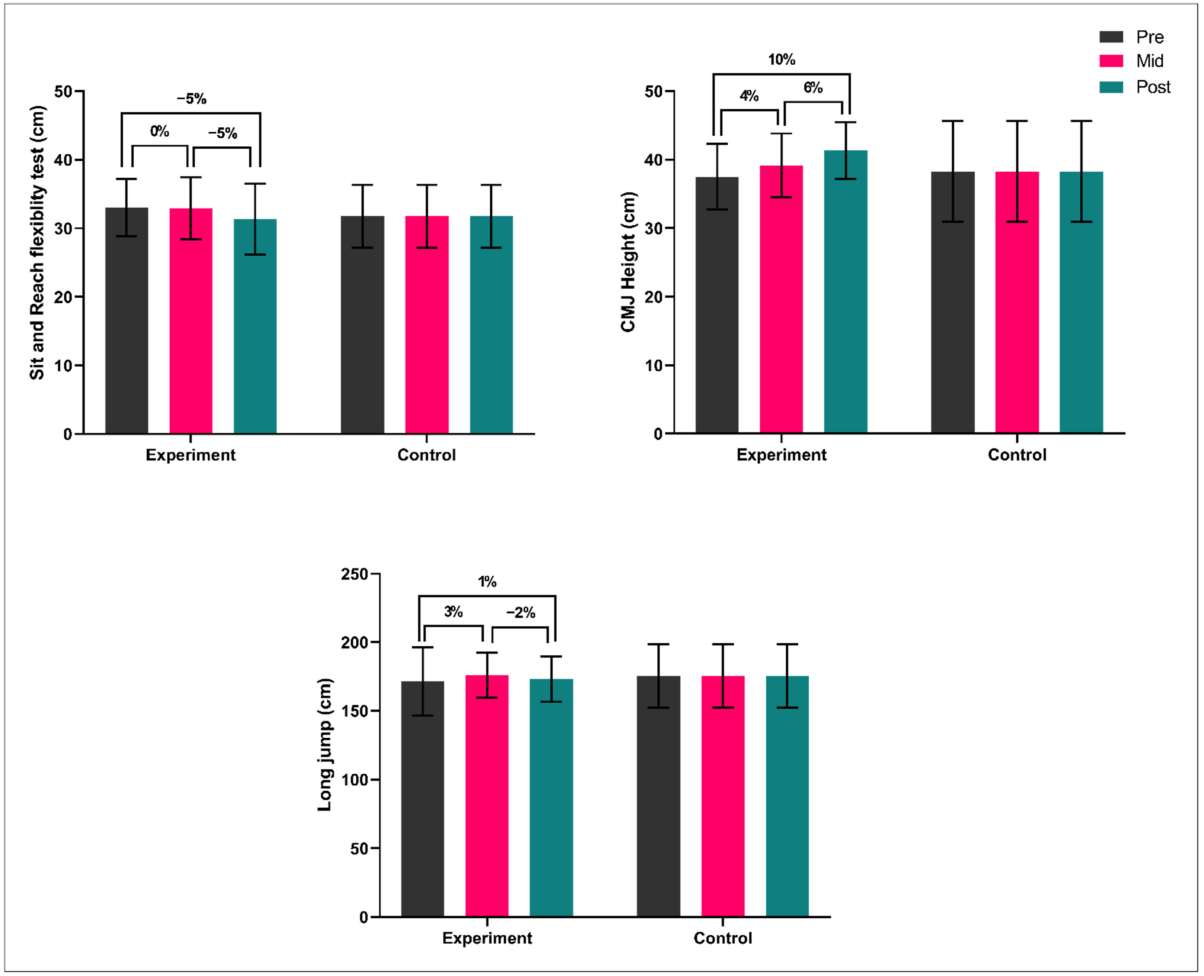
Descriptive statistics, within-group variations of means of flexibility, CMJ and LJ parameters.

**Figure 3:**
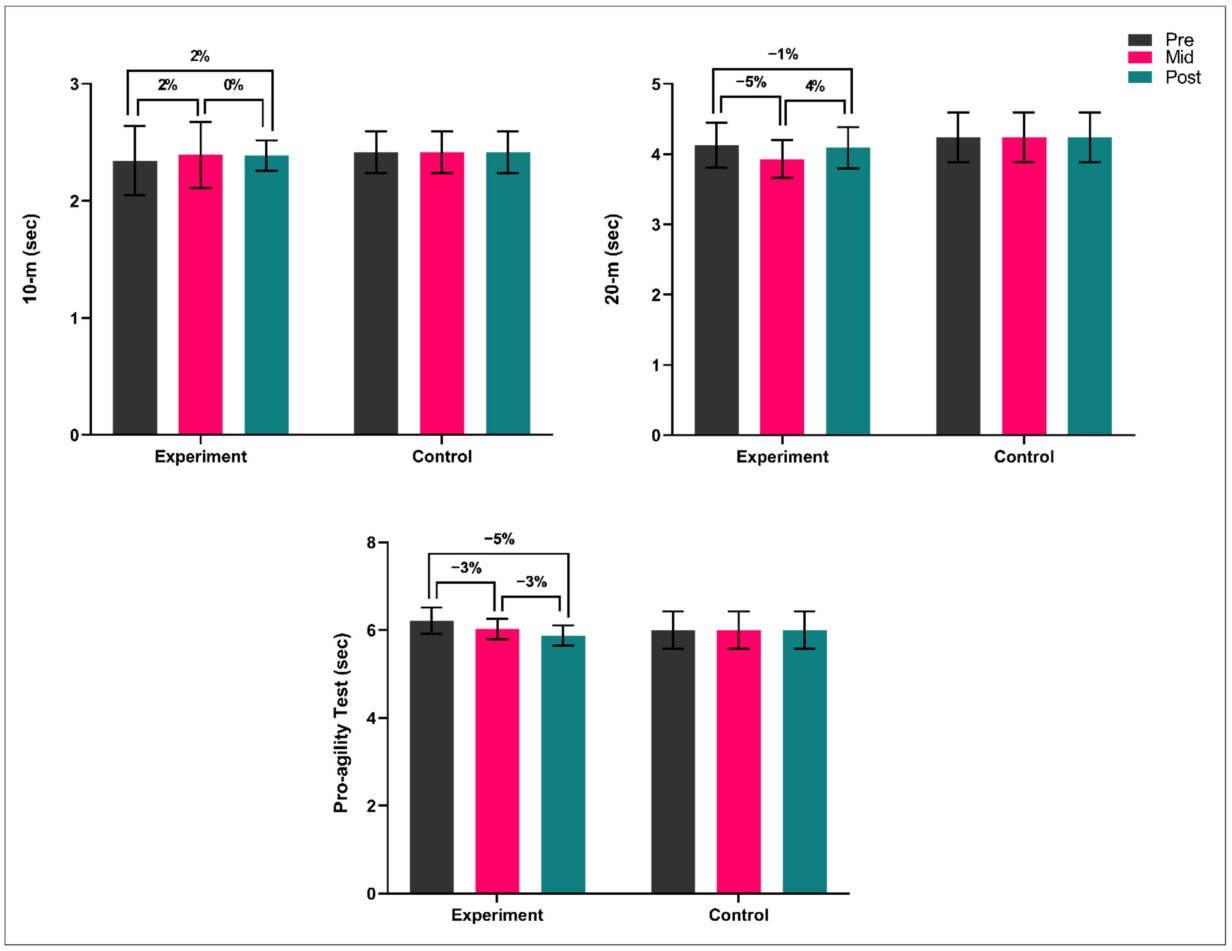
Descriptive statistics, within-group variations of means of 10 m, 20 m sprint time and pro-agility test (COD).

**Figure 4:**
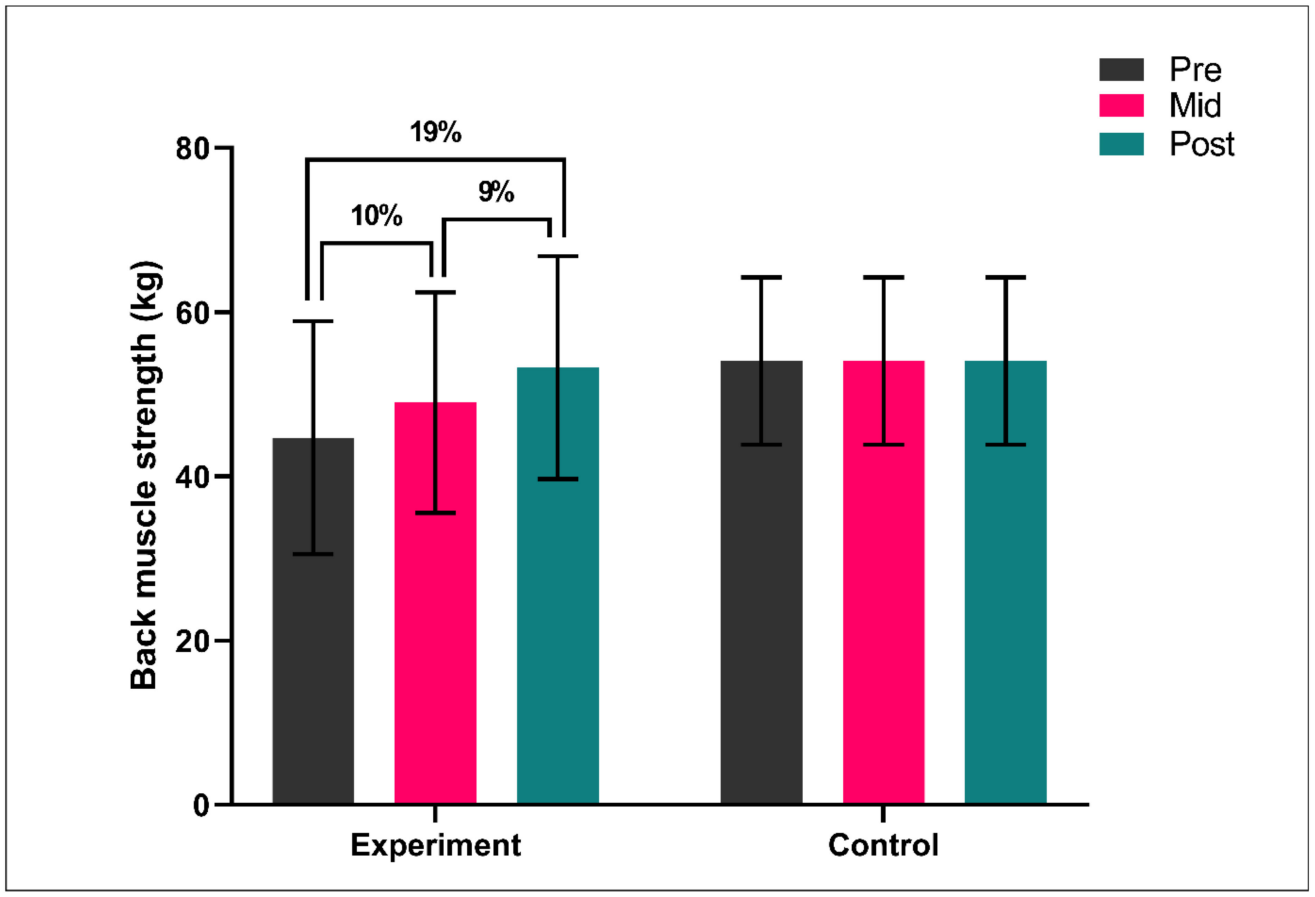
Descriptive statistics, within-group variations of means of back muscle strength (BMS).

**Figure 5:**
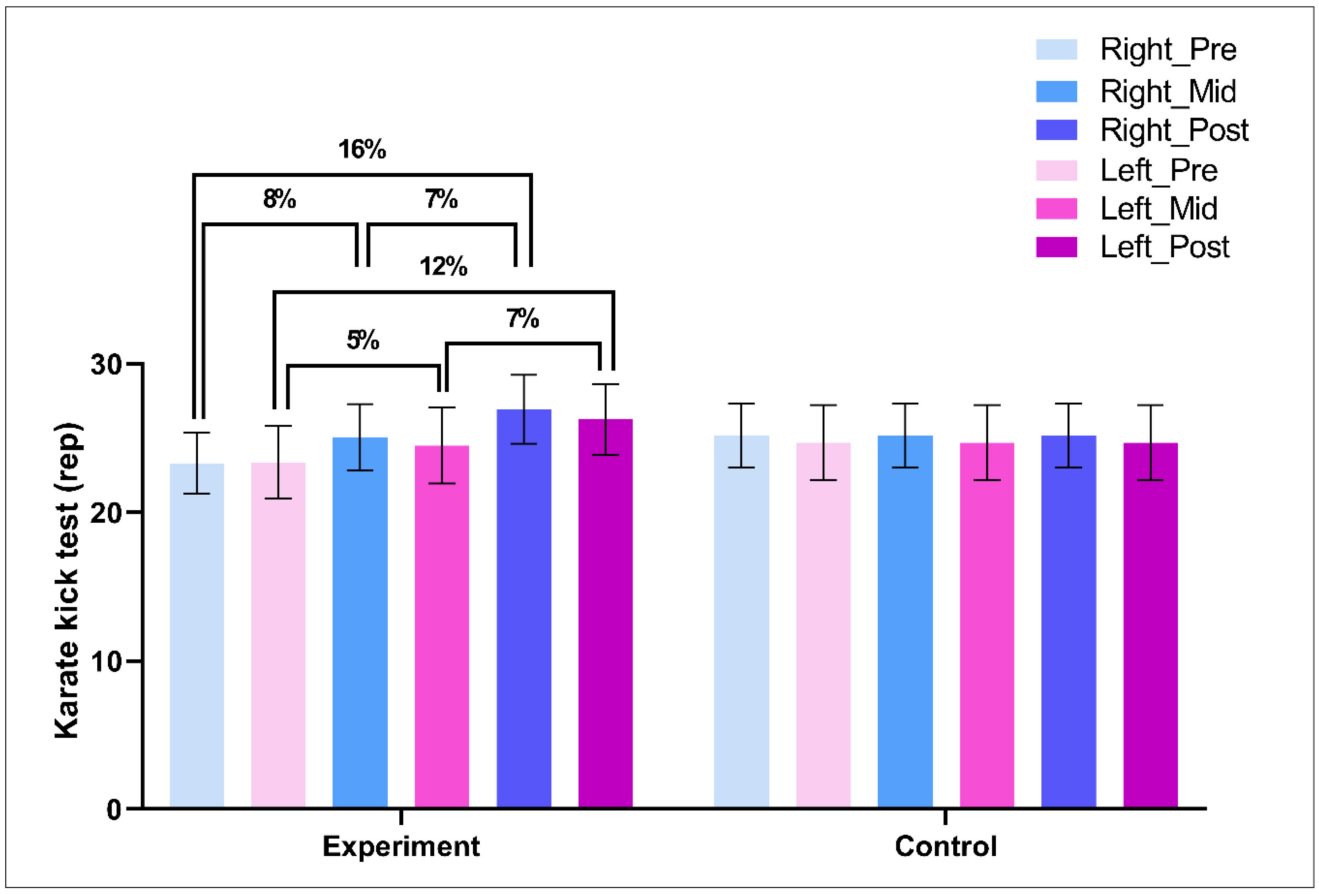
Descriptive statistics, within-group variations of means of karate kick test (KKT).

Figure 1 presents the within- and between-group variations FET, BET and TFT tests. Figure 2 presents the within- and between-group variations for CMJ and LJ tests. Figure 3 presents the within- and between-group variation for 10 m sprint time, 20 m sprint time and pro-agility tests. Figure 4 presents the within- and between-group variations for BMS tests. Figure 5 presents the within- and between-group variations for KKT tests.

## 4. Discussion

The CST group had significant benefits after intervention in the FET, BET and LMT, 20 m sprint and KKT in comparison to those not exposed to CST. However, results revealed no significant differences between experimental and control group on the flexibility, COD, CMJ and LJ, BMS, and 10 m sprinting. The within-experimental group changes after training intervention revealed significant improvements in flexibility, COD, CMJ, BMS, 20 m sprint and KKT, while control groups had significantly decreased performance in FET, BET, LMT, BMS and KKT for the right side. 

CST is commonly defined as the ability to generate and maintain force, while core stability can be considered as the ability to actively or passively stabilize the lumbopelvic region to maintain appropriate trunk and hip posture and static and dynamic balance [42]. Thus, core stability can be linked to the ability to maintain core control while applying core strength [32]. Although a belief that isolated or dedicated CST can be more effective for improving both core strength and core stability, it seems that such a hypothesis is not confirmed regarding the muscle activity while performing exercises [3]. In a brief review, it was disclosed that multijoint free weight exercises imply greater lumbar multifidus electromyographical activity than core exercises, while the transverse abdominis are similarly activated by CST and free weights [43]. Moreover, adaptations resulting from targeted core stability seem to provide marginal benefits for athlete performance, considering a systematic review performed about the topic [27]. 

Such a synthesis supports the evidence obtained in the current research. Those who experienced the CST programme did not significantly benefit when compared to those not following the training programme regarding the main physical performance variables (e.g., flexibility, COD, CMJ, BMS, LJ and 10 m sprint). Considering that the core stabilizes and allows for energy transfer from the lower limb to the remaining body due to lower limb attachment at the hip joint, it would be expected to observe benefits for activities such as running, jumping or back squat [44,45]. However, in our research, none of the jumping (either vertical or horizontal), back squat, short sprint, and COD were significantly improved by CST in comparison to the control group. Apparently, this can be associated with the trainability of the participants. It seems that untrained populations can benefit from CST; however, those with experience in training may not significantly benefit from hip flexor strength training [43]. Possibly, body exercises focused on the core may not be impactful enough for providing a significant stimulus for adaptation. Thus, possibly, CST may not be as useful as expected for meaningful impact and complex movements such as running, jumping or produce force. 

Although there was an absence of meaningful benefits of CST in most athletic performance outcomes, a significant beneficial impact on the FET, BET, LMT and KKT was observed in comparison to the control group. Those not participating in the CST significantly dropped in performance for FET, BET, LMT and KKT. Since in most typical sport activities the core acts as a stabilizer of the spine and pelvis rather than producing gross movements of the trunk and hip [46,47], it was anticipated that a dedicated CST may meaningfully impact core endurance testing. As an example, the internal obliques seem to be highly recruited in core stability exercises rather than with free weights, which [46] can offer a view in which CST may provide some interesting effects for core endurance tests. Moreover, since karate kicks involve some level of peak force to create an inertial mass in the core for limb muscles to pry against to initiate limb motion [48], it seems that core-centred training can provide additional benefits for karate kicks. 

Naturally, we have only focused on physical fitness outcomes in karate. However, CST may have an impact on other factors. A previous study revealed a decrease in risk factors such as for the anterior cruciate ligament (ACL) while performing jumping movements after performing CST [49]. Moreover, the absence of improvements can be caused by the type of sport itself. As an example, in swimming, CST produced significant improvements in endurance, swim record, stability and anaerobic peak power [50]. The same evidence of advantage in different physical fitness measures was found in handball [51].

The current research has some limitations. The specific karate training was not monitored, which does not allow for how the control and experimental groups trained besides for the core intervention. This may justify some of the results of the study. Moreover, the level of intensity of the exercises is one of the flaws that is found in most studies conducted on CST, since it is hard to individualize the stimulus. Future studies should consider analysing the effects of different intensity levels while applying core training and should consider all the exercises performed in specific karate training, aiming to analyse the impact of that on the final outcomes. 

Despite the limitations, this study presents some evidence that CST in karate can enhance improvements in core endurance and kick movements, although not significantly impact the remaining physical qualities. Possibly, the core strength and stabilization training should be performed combined with other strength exercises (as free weight), aiming to maximize the effects of both. 

## 5. Conclusions

The current parallel study design revealed that CST in karate provided centred benefits on core endurance and kicking movements, while not significantly impacting other physical qualities compared to those not experiencing such type of training. CST should be considered as an element to be introduced in a combined strength training routine using different methods which may benefit athletic performance of karate athletes. As for scientific evidence and for practical implication, it is possible to suggest that CST in karate athletes is not significantly advantageous for improving most physical fitness outcomes. Thus, it can be only used as a compliment.

## Figures and Tables

**Table 1:** Training programme design.

	Weeks								Core Strength Training Programme Details	Sets	Reps and Time (s)	Rest (s) (Exercises/Sets)
	1	2	3	4	5	6	7	8				
**CG**	Sport-specific program								Push up Abdominal crunch Vertical leg crunch Jack knife Supine knee side-to-side Reverse crunch Plank jack	3	12	90 s/45 s
**EG**	Sport-specific program + Core strength training program											
									Superman Cat–camel stretch	3	30 s	

CG: control group, EG: experimental group.

**Table 2:** Phases of the training programs.

		Sunday	Monday	Tuesday	Wednesday	Thursday	Friday	Saturday
1st wk	**CG**	Visit 1	Visit 2	SSp	SSp	SSp	SSp	SSp
	**EG**			SSp + CSTp	SSp	SSp + CSTp	SSp	SSp + CSTp
2nd wk	**CG**	Rest day	SSp	SSp	SSp	SSp	SSp	Rest day
	**EG**		SSp + CSTp	SSp	SSp + CSTp	SSp	SSp + CSTp	
3rd wk	**CG**	Rest day	SSp	SSp	SSp	SSp	SSp	Rest day
	**EG**		SSp + CSTp	SSp	SSp + CSTp	SSp	SSp + CSTp	
4th wk	**CG**	Rest day	SSp	SSp	SSp	SSp	SSp	Rest day
	**EG**		SSp + CSTp	SSp	SSp + CSTp	SSp	SSp + CSTp	
5th wk	**CG**	Visit 3	Visit 4	SSp	SSp	SSp	SSp	SSp
	**EG**			SSp + CSTp	SSp	SSp + CSTp	SSp	SSp + CSTp
6th wk	**CG**	Rest day	SSp	SSp	SSp	SSp	SSp	Rest day
	**EG**		SSp + CSTp	SSp	SSp + CSTp	SSp	SSp + CSTp	
7th wk	**CG**	Rest day	SSp	SSp	SSp	SSp	SSp	Rest day
	**EG**		SSp + CSTp	SSp	SSp + CSTp	SSp	SSp + CSTp	
8th wk	**CG**	Rest day	SSp	SSp	SSp	SSp	SSp	Rest day
	**EG**		SSp + CSTp	SSp	SSp + CSTp	SSp	SSp + CSTp	
9th wk	**CG**	Visit 5	Visit 6	Routine training period				
	**EG**							

**CG:** control group, **EG:** experimental group, **SSp:** sport-specific program, **CSTp:** core strength training program.

**Table 3:** Participant characteristics.

	Experimental Group			Control Group		
	Female (*n* = 11)	Male (*n* = 5)	Total (*n* = 16)	Female (*n* = 6)	Male (*n* = 7)	Total (*n* = 13)
**Age (year)**	12.8 ± 0.83	12.72 ± 0.78	12.75 ± 0.77	13.25 ± 0.95	12.88 ± 0.92	13 ± 0.91
**Height (cm)**	150.2 ± 11.07	153.18 ± 10.16	152.25 ± 10.18	154.25 ± 11.75	152.33 ± 10.78	152.92 ± 10.62
**Weight (kg)**	43.2 ± 8.19	43.18 ± 10.28	43.18 ± 9.40	45.25 ± 5.25	41.44 ± 9.56	42.61 ± 8.44
**Experience (year)**	4.36 ± 0.50	4.45 ± 0.52	4.43 ± 0.51	4.36 ± 0.50	4.63 ± 0.51	4.31 ± 0.47

No differences were observed between experimental and controls. All comparisons had *p* values ≥ 0.05.

**Table 4:** Descriptive statistics (mean and standard deviation), within- and between-group analysis.

	Experimental (Within Pre, Mid and Post Time Group Analysis)		Control (Within Pre, Mid and Post Time Group Analysis)		Between-Group Differences (Pre)		Between-Group Differences (Mid)		Between-Group Differences (Post)	
Variables	*p*	$\eta_{p}^{2}$	*p*	$\eta_{p}^{2}$	*p*	d	*p*	d	*p*	d
**FET (sec)**	≤0.001	0.836	0.487	0.021	0.119	0.601	0.010	−1.036	≤0.001	−2.802
**BET (sec)**	≤0.001	0.773	0.674	0.055	0.774	−0.108	0.002	−1.275	≤0.001	−2.946
**Right LMT (sec)**	≤0.001	0.804	0.643	0.015	0.651	0.171	0.006	−1.112	≤0.001	−2.190
**Left LMT (sec)**	≤0.001	0.864	0.532	0.017	0.622	−0.186	≤0.001	−1.646	≤0.001	−3.096
**Flexibility (cm)**	0.002	0.335	0.024	0.211	0.434	−0.296	0.012	−1.009	0.700	−0.145
**Pro-agility (sec)**	≤0.001	0.729	0.613	0.040	0.126	−0.590	0.483	−0.266	0.654	0.169
**CMJ (cm)**	≤0.001	0.660	0.778	0.021	0.732	0.129	0.835	−0.078	0.172	−0.524
**BMS (kg)**	≤0.001	0.786	0.312	0.085	0.057	0.743	0.758	0.116	0.834	−0.079
**Horizontal jump (cm)**	0.319	0.073	0.822	0.016	0.650	0.171	0.790	0.100	0.632	0.181
**10 m (sec)**	0.776	0.017	0.055	0.215	0.442	0.291	0.266	−0.424	0.200	−0.490
**20 m (sec)**	0.033	0.204	0.270	0.103	0.384	0.331	0.006	1.126	0.021	0.912
**Right kick (rep)**	≤0.001	0.886	0.042	0.232	0.026	0.877	0.285	−0.407	0.006	−1.121
**Left kick (rep)**	≤0.001	0.752	0.167	0.138	0.167	0.530	0.616	−0.189	0.020	−0.927

**FET:** flexor endurance test; **BET:** back extensor test; **LMT:** lateral musculature test; **Rep:** repetitions; **Sec:** seconds. Statistical significance was set to *p* < 0.05.

## Data Availability

The datasets used and/or analysed during the current study are available from the corresponding author upon reasonable request.
